# Enzyme‐Digested Edible Bird's Nest Promotes NGF‐Induced Differentiation of PC12 Cells by Activating the TrkA‐ERK1/2‐CREB‐BDNF Signaling Pathway

**DOI:** 10.1002/fsn3.72419

**Published:** 2026-10-06

**Authors:** Wen Zhang, Xintong Wang, Huiqing Zhu, Xiaoxian Lin, Rongzi Li, Man Yuan, Baocai Xu, Dongliang Wang, Wei Xiong, Yixuan Li

**Affiliations:** ^1^ Key Laboratory of Precision Nutrition and Food Quality, Department of Nutrition and Health China Agricultural University Beijing China; ^2^ School of Food and Biological Engineering Hefei University of Technology Hefei Anhui China; ^3^ Hebei Edible Bird's Nest Fresh Stew Technology Innovation Center Langfang Hebei China; ^4^ Food Laboratory of Zhongyuan Luohe Henan China

**Keywords:** edible bird's nest, neuronal differentiation, PC12 cells, TrkA‐ERK1/2‐CREB‐BDNF

## Abstract

The neuroprotective properties of edible bird's nest (EBN) are well established; however, its effects on neuronal growth remain unclear. In this study, we investigated the effects of enzyme‐digested EBN (EBND) on neurogenesis in the PC12 neuronal cell line. Although treatment with EBND alone did not significantly promote the differentiation of PC12 cells, it increased neurite outgrowth and neurofilament expression when co‐administered with nerve growth factor (NGF). Moreover, EBND significantly augmented the NGF‐induced phosphorylation of TrkA and ERK1/2, which in turn activated CREB and increased brain‐derived neurotrophic factor (BDNF) expression. Notably, these effects of EBND were diminished by the TrkA inhibitor GW441756. Taken together, these findings suggest that combined treatment with EBND and NGF enhances neurogenesis in PC12 cells by activating the TrkA‐ERK‐CREB‐BDNF signaling pathway.

## Graphical abstract

EBND alone upregulates neurofilament expression but does not induce neurite outgrowth in PC12 cells. With suboptimal NGF, EBND synergistically promotes neuronal differentiation via the TrkA‐ERK1/2‐CREB‐BDNF pathway. This effect is blocked by the TrkA inhibitor GW441756, confirming a TrkA‐dependent mechanism.

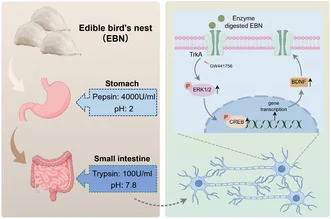

## 1 Introduction

Edible bird's nest (EBN) is a widely‐consumed functional food in Asian countries, and is derived from the salivary secretions of swiftlets commonly found in countries such as Thailand, Indonesia, and Malaysia. EBN consumption in Asia dates back to the Tang dynasty (681 ad), when it was revered as a valuable tonic, and it has been called the “Caviar of the East” (Dai et al. 2021; Wong 2013; Marcone 2005). EBN is considered a beneficial dietary supplement and therapeutic agent in Traditional Chinese Medicine, specifically for a range of respiratory and digestive ailments. EBN has been reported to enhance immune function, ameliorate signs of aging, alleviate symptoms of asthma and cough, and mitigate stomach ulcers (Loh et al. 2022).

Neurogenesis encompasses the intricate process of neural stem cell (NSC) proliferation and subsequent differentiation into mature neurons. In the brain, neurogenesis is essential for neural plasticity, the maintenance of brain homeostasis and cognitive function (Poulose et al. 2017). Adult neurogenesis is adversely affected by several lifestyle factors, including unhealthy diets and alcohol addiction, as well as biological factors such as aging, oxidative stress, and neuroinflammation (Poulose et al. 2017). Perturbed adult neurogenesis has been implicated in various neurodegenerative diseases, including Alzheimer, Huntington, and Parkinson diseases, which are characterized by progressive neuronal loss and behavioral and memory impairments (Terreros‐Roncal et al. 2021). Recent studies show that enhancing adult neurogenesis may improve cognitive function (Cui et al. 2023). Therefore, promoting neurogenesis using specific dietary factors may be an effective strategy for delaying or preventing neurodegenerative diseases.

An important nutrient in EBN is sialic acid (SA), which has been shown to have neuroprotective properties, promote brain development, and enhance memory (Wang et al. 2007). Sialic acid is reported to improve intelligence and brain function in children by enhancing synaptic pathways and ganglioside distribution (Wang 2009). In addition to SA, EBN also contains lactoferrin, yolk transferrin, N‐acetylgalactosamine, and epidermal growth factor, which have neuroprotective effects and contribute to healthy brain development (Chu et al. 2023). We previously showed in animal studies that maternal administration of EBN improves spatial learning memory in offspring by promoting hippocampal neurogenesis (Zhu et al. 2023). However, the mechanisms underlying the neuroprotective actions of EBN remained unclear.

Here, our study was focused on investigating the impact of EBN on neuronal differentiation (an important stage of neurogenesis). The PC12 pheochromocytoma cell line is a neural cell line derived from an adrenal tumor and has been widely used to study neuronal differentiation. PC12 cells can be induced by nerve growth factor (NGF) to differentiate into cells with typical neuronal phenotypes and extend neurites (Fujita et al. 1989; Spillane et al. 2013). PC12 cells are therefore frequently used to study neuronal differentiation. The assessment of neuronal differentiation in PC12 cells involves measuring the length of neurites, which is widely used as a morphological marker of differentiation. In addition, neurofilament expression can also be used as a biomarker of differentiation in PC12 cells (Yan et al. 2017). In this study, we investigate the effect of enzyme‐digested EBN (EBND) on neuronal differentiation in PC12 cells, assessed by measuring neurite length and neurofilament expression. Furthermore, we examine the underlying mechanisms of action of EBND.

## 2 Materials and Methods

### 2.1 Reagents and Equipment

#### 2.1.1 Reagents

Pepsin (≥ 2500 U/mg), trypsin (≥ 250 U/mg), and the Cell Counting Kit‐8 (CCK‐8) were purchased from Solarbio Science & Technology Co. Ltd. (Beijing, China). Nerve growth factor (NGF) was obtained from Sigma‐Aldrich. The TrkA inhibitor GW441756 was sourced from MedChemExpress (USA). RPMI‐1640 medium for cell culture was purchased from Procell Life Science & Technology Co. Ltd. (Wuhan, China), while fetal bovine serum (FBS) and penicillin–streptomycin solution were acquired from Gibco. Reagents for cell fixation and permeabilization, including 4% paraformaldehyde and rapid permeabilization solution, were obtained from Beyotime Biotechnology (Shanghai, China).

#### 2.1.2 Equipment

Multifunctional Microplate Detector (Synergy HTX. Biotek, USA); CO_2_ cell incubator (51026280, Thermo Fisher, USA); Fluorescence Microscope (DM6B, Leica, Germany); Cellular Ultrasonic Disruptor (FS‐450S, Shanghai Shengxie Ultrasonic Instrument Co. Ltd.); Organizer Crusher, Electrophoresis apparatus (1645050, Bio‐Rad, USA); Gel Imaging System (ChemiScope 6100, Shanghai Qinxiang Scientific Instrument Co. Ltd.).

### 2.2 Preparation of EBN Samples and SA

The EBND was prepared as follows: The macronutrient composition of the EBN material has been characterized in our previous study (Zhu et al. 2023). First, the EBN soaked in distilled water was heated in water at 95°C for 10–25 min. 100 mL of the EBN solution was then removed and shaken on a shaker (50 rpm) for 3 h at 55°C. Hydrochloric acid was used to adjust the pH to 2.0, and pepsin (4000 U/mL) was added. The samples were then shaken for 2 h and boiled for 20 min (to inactivate pepsin). Subsequently, the pH was adjusted to 7.8, and trypsin (100 U/mL) was added. The digested solution was subsequently placed on a shaker and incubated at 37°C for 2 h to simulate small bowel conditions. The reaction was then terminated by boiling for 30 min, and upon cooling, the digested sample was lyophilized for subsequent experiments. The dried EBN used was produced in Malaysia and purchased from Beijing Xiaoxiandun Biotechnology Co. Ltd. (Beijing, China). The SA standards used in the experiments were > 97% pure and were purchased from Solarbio (A8871, Beijing, China).

### 2.3 Cell Culture

Undifferentiated PC12 cells (CL‐0412, Procell, Wuhan, China) were cultured in RPMI‐1640 medium supplemented with 15% horse serum, 6% fetal bovine serum, and 1% penicillin/streptomycin. The cells were maintained under standard conditions in a humidified CO_2_ incubator at 37°C, with the medium replaced every other day with fresh medium. All culture reagents were purchased from Procell Life Science and Technology (Procell, Wuhan, China).

### 2.4 Cell Viability Assays

Cell viability was assessed using the Cell Counting Kit‐8 (CCK‐8) assay. PC12 cells were initially seeded into 96‐well plates at a density of 5 × 10^3^ cells/well and allowed to grow for 24 h. Subsequently, the cells were treated with EBND for 24 h. Following treatment, 10 μL of CCK‐8 solution was added to each well and incubated in the dark at 37°C for 2 h. The optical density (OD) values were measured at 450 nm using an enzyme labeler (Thermo, USA). Relative cell viability was expressed as a percentage of the control. Cell viability analysis was performed to determine the safe concentration range of the EBN extract, which was tested at concentrations ranging from 0 to 1000 μg/mL. The extracts did not induce cell proliferation or death within this concentration range.

### 2.5 Analysis of Neurite Outgrowth of PC12 Cells

PC12 cells were seeded onto 12‐well plates (P2100, Solarbio, Beijing, China) at a density of 1 × 10^4^ cells/mL and incubated for 24 h. The plates were pre‐coated with Poly‐L‐Lysine to enhance cell attachment. Subsequently, cells were exposed to EBND with or without the addition of a low concentration (1.5 ng/mL) of nerve growth factor (NGF), and the medium was replaced every other day. On day 7, PC12 cells were photographed using a light microscope (Leica DM6B, Germany). Cells were classified as differentiated if the length of at least one neurite exceeded the diameter of the corresponding cell body. Cells were analyzed for neurite length using Image J software (Version 7.0). At least 100 cells in three typical fields were chosen randomly from each well to calculate the number of differentiated cells and the average length of the neurites. At least three independent experiments were performed. Normalization of neurite measurements. To ensure comparability across different experimental repeats and wells, neurite measurements were normalized as follows: Average neurite length: For each dish, use Image J to measure the neurite length of all measurable differentiated cells. Calculate the average neurite length for each dish. Then, average the values of the same experimental group from three independent repeated experiments (dishes). The final data are presented in the form of the average of these average values ± standard error.

### 2.6 Immunostaining for Neurite Outgrowth Assay

After pharmacological intervention, we fixed PC12 cell morphology using 4% paraformaldehyde (P0099, Beyotime, Shanghai, China). Subsequently, the cells were permeabilized with rapid permeabilization solution for 20 min at room temperature, followed by overnight incubation at 4°C with the β‐III‐tubulin (ab18207, 1:1000, Abcam, UK) primary antibody. Then, the cells were incubated with goat anti‐rabbit secondary antibody conjugated to Alexa Fluor 488 (Beyotime, Shanghai, China) for 1 h at room temperature. DAPI was used for nuclear counterstaining. Images were captured using an Airyscan confocal microscope (ZEISS, Jena, Germany).

### 2.7 Western Blotting Assay

PC12 cells were cultured in 12‐well plates coated with Poly‐L‐Lysine, with an initial seeding density of 2 × 10^4^ cells/well. Thereafter, the cells were treated with medium containing the drug. On day 7, PC12 cells were lysed with RIPA solution (P0013C, Beyotime, Shanghai, China), and total protein concentration was determined using the BCA Kit (P0012, Beyotime, Shanghai, China). Equal quantities of protein were employed for the Western blot analysis, following the methodology described earlier (Zhu et al. 2023). Finally, the blots were developed using a luminol‐based enhanced chemiluminescence kit. The primary antibodies, anti‐NF68 (ab223343, 1:1000), anti‐NF160 (ab254348, 1:1000), anti‐NF200 (ab207176, 1:1000), and anti‐β‐actin (ab8226, 1:1000), were purchased from Abcam (Cambridge, UK). The primary antibodies, anti‐TrkA (2510S, 1:1000), anti‐p‐TrkA (4619 T, 1:1000), anti‐ERK1/2 (4695 T, 1:1000), anti‐p‐ERK1/2 (4370 T, 1:1000), anti‐CREB (9197 T, 1:1000), and anti‐p‐CREB (9198S, 1:2000), were purchased from Cell Signaling Technology (Danvers, MA, USA).

### 2.8 Analysis of Signaling Pathways Involved in PC12 Cell Differentiation

To examine whether EBND promotes neurogenesis in PC12 cells by activating the TrkA‐ERK1/2‐CREB‐BDNF pathway, we used an inhibitor of the TrkA receptor (GW441756) (MedChemExpress, USA). The inhibitor was prepared in DMSO and stored at −20°C in the dark as stock solutions at a concentration of 10 mM. GW441756 was diluted in complete medium before use to a final concentration of 100 nM. After a pre‐incubation period of 1 h with the inhibitor, the cells were treated with varying doses of EBND for 48 h. Afterwards, total cellular proteins were collected and the levels of neurofilament protein were measured using western blot.

### 2.9 Statistical Analysis

All data were expressed as mean ± standard error of the means (SEM). Statistical analyses were performed using GraphPad Prism version 9.5.1. A one‐way analysis of variance (ANOVA) was applied for intergroup statistical comparison, followed by Dunnett's multiple comparisons post hoc test. Statistical significance levels were classified as: *p < 0.05, **p < 0.01, ***p < 0.001.

## 3 Results

### 3.1 Treatment of PC12 Cells With EBND Alone Fails to Induce Neurite Growth, but Increases Expression of Neurofilament Proteins

The EBND concentrations (10, 50, and 100 μg/mL) used in this study were selected based on the findings from our previous animal experiments (Zhu et al. 2023) and the CCK‐8 cell viability assay (Figure S1 and Table S1). As shown in Figure S1 and Table S1, EBND exhibited no cytotoxicity toward PC12 cells within the concentration range of 1–1000 μg/mL after 24 h of treatment, with a gradual increase in cell viability observed at 1–100 μg/mL. Therefore, concentrations of 10, 50, and 100 μg/mL were selected for subsequent differentiation experiments. To evaluate the impact of EBND on PC12 cell differentiation, we used microscopy to observe cell morphology. Based on quantitative morphological analysis, EBND treatment did not significantly affect the growth of neurites (Figure 1A–C). We also investigated the impact of EBND on the levels of neurofilament expression, a definitive biomarker of neuronal differentiation. After 7 days of incubation with EBND, all three neurofilament proteins were slightly upregulated (Figure 2). Among the three examined neurofilaments, NF68 exhibited higher expression compared to NF160 and NF200. Compared with the control group, NF68 expression more than tripled (*p* < 0.001), and the levels of NF160 and NF200 were increased at least 2‐fold after exposure to EBND at a concentration of 100 μg/mL (*p* < 0.001) (Figure 2A,B). A full summary of the one‐way ANOVA statistics, including *F*‐values and *p*‐values for all experimental groups, is provided in Table S2.

**FIGURE 1:**
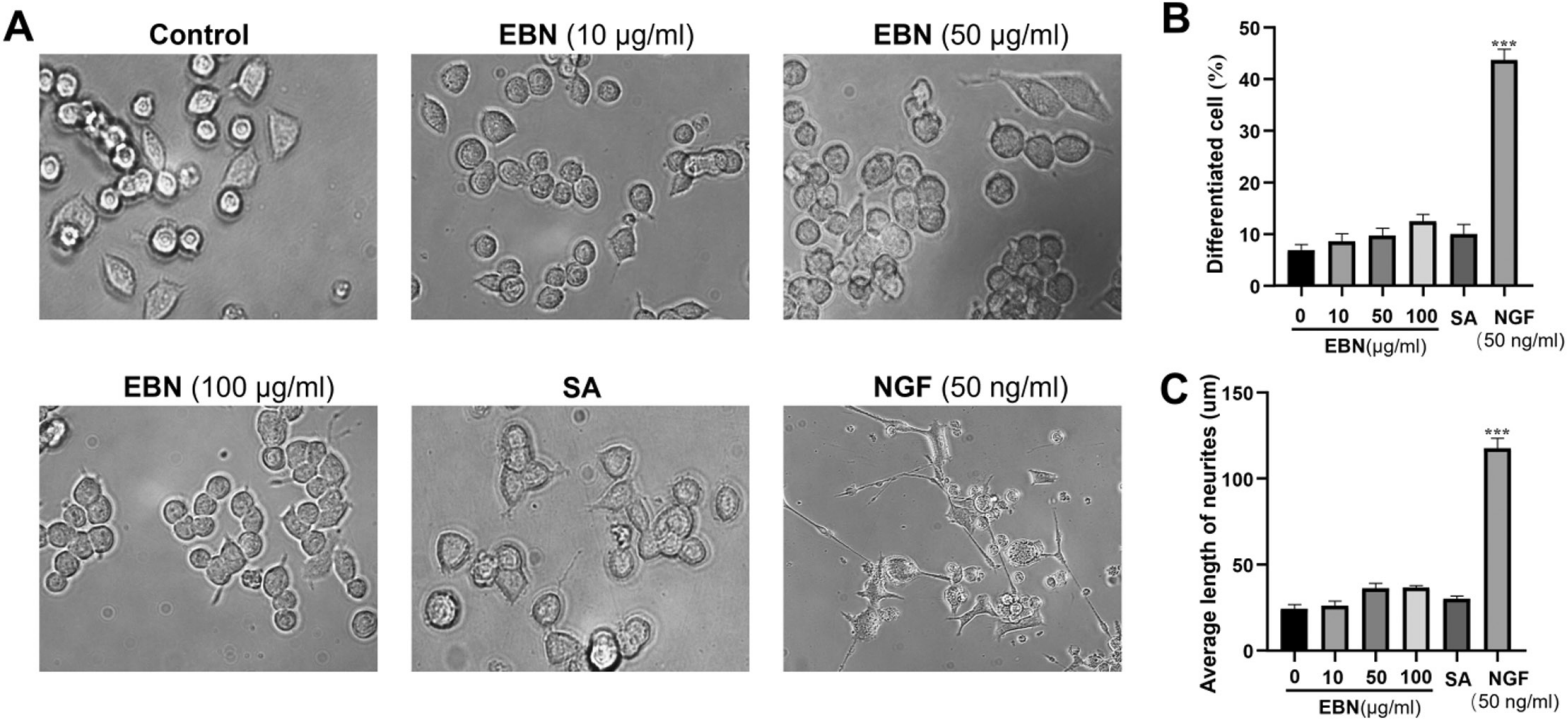
EBND does not induce neurite growth in PC12 cells. (A) A representative micrograph illustrating cell differentiation observed on day 7 post‐dosing. (B, C) Quantitative results of number of differentiated cells and average neurite lengths. *n* ≥ 3, (***p < 0.001).

**FIGURE 2:**
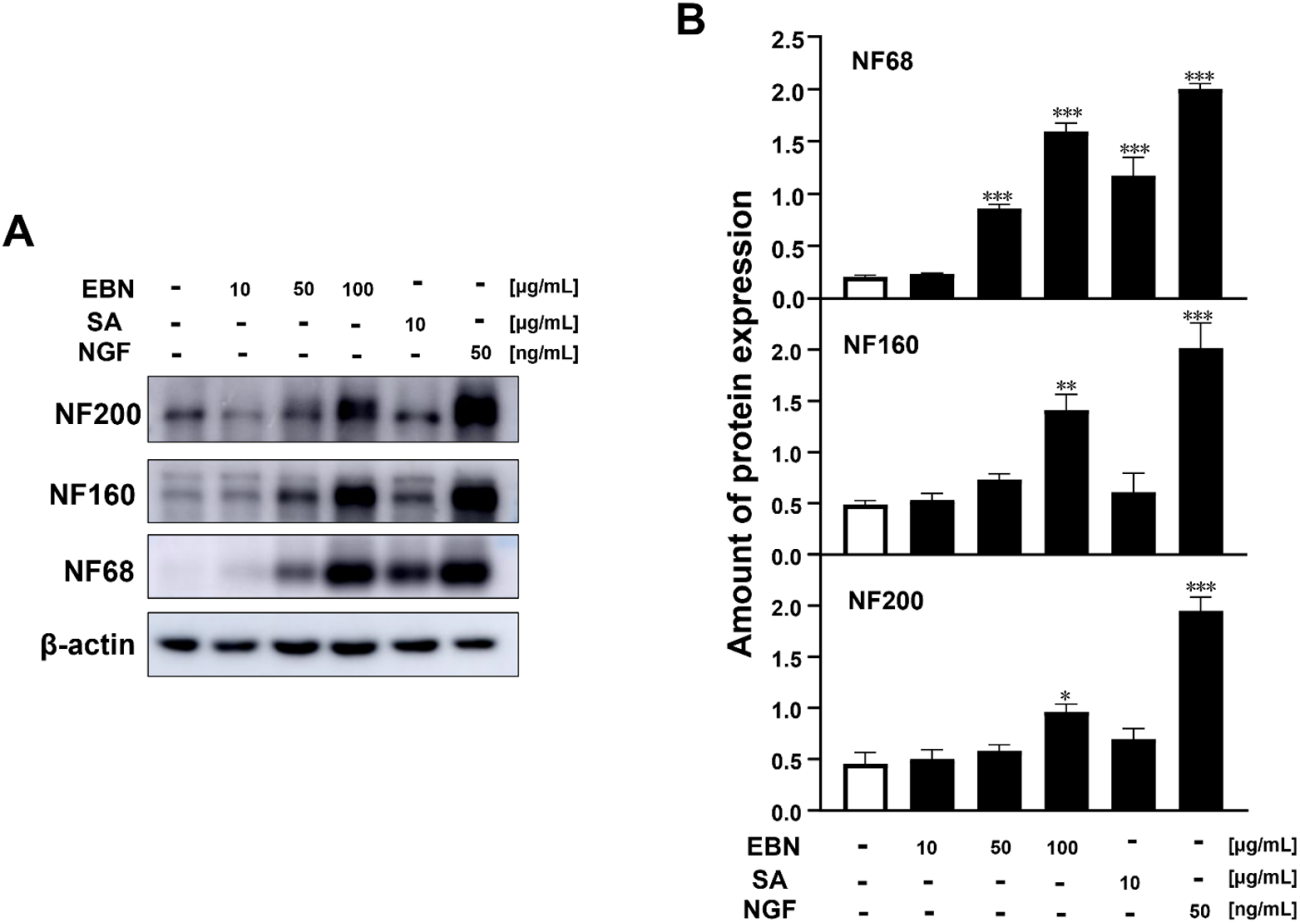
EBND stimulates neurofilament expression on PC12 cells. (A, B) PC12 cells were induced with 10, 50, or 100 μg/mL EBND and SA, respectively, and 50 ng/mL NF was used as a positive control, and protein expression levels of neurofilaments NF68, NF160, and NF200 were detected using western blot. Data are presented as mean ± SEM, *n* ≥ 3. (*p < 0.05, **p < 0.01, ***p < 0.001).

### 3.2 EBND Enhances NGF‐Induced Neurite Outgrowth in PC12 Cells

Although EBND significantly increased the expression levels of neurofilament proteins in PC12 cells, it did not markedly promote neurite outgrowth when administered alone. NGF is critical for the differentiation of PC12 cells, with its absence resulting in negligible differentiation of these cells (Zhu et al. 2016). Therefore, we examined whether EBND could enhance NGF‐stimulated neurite growth. When cells were cultured under low‐serum conditions, NGF at a concentration of 1.5 ng/mL failed to induce differentiation (Figure 3A,B). Therefore, an NGF concentration of 1.5 ng/mL was used in combination with EBND for the co‐treatment experiments. An NGF concentration of 50 ng/mL was used as a positive control because of its demonstrated ability to induce neurite outgrowth in PC12 cells by activating the TrkA receptor (Xiong Gao et al. 2021). In the neurite growth assay, cells in which the length of the neurite was greater than the diameter of the cell body were defined as differentiated cells. The differentiation rates of PC12 cells treated with 10 μg/mL, 50 μg/mL and 100 μg/mL EBND were 22.8% (*p* = 0.12), 31.2% (*p =* 0.002) and 42.7% (*p* < 0.001), respectively, which were significantly higher than those of cells cultured with control medium containing 1.5 ng/mL NGF (Figure 3A,B). As the EBND concentration increased from 10 μg/mL to 100 μg/L, the mean neurite length increased in tandem and was consistent with the percentage of cells with neurites (10 μg/mL, *p* = 0.797; 50 μg/mL, *p <* 0.001; 100 μg/mL, *p* < 0.001) (Figure 3C). In biochemical assays, there was an increase in the expression levels of all three neurofilament subunits. Compared with the 1.5 ng/mL NGF group, cells treated with 10 μg/mL EBND exhibited a great increase, of 99.2% (*p* = 0.03). and 34.5% (*p* = 0.4), in the expression levels of NF160 and NF200, respectively (Figure 4A,B). Furthermore, in cells treated with 50 μg/mL and 100 μg/mL EBND, there was a 2–3‐fold increase in the levels of the three neurofilament proteins (*p* < 0.001) (Figure 4).

**FIGURE 3:**
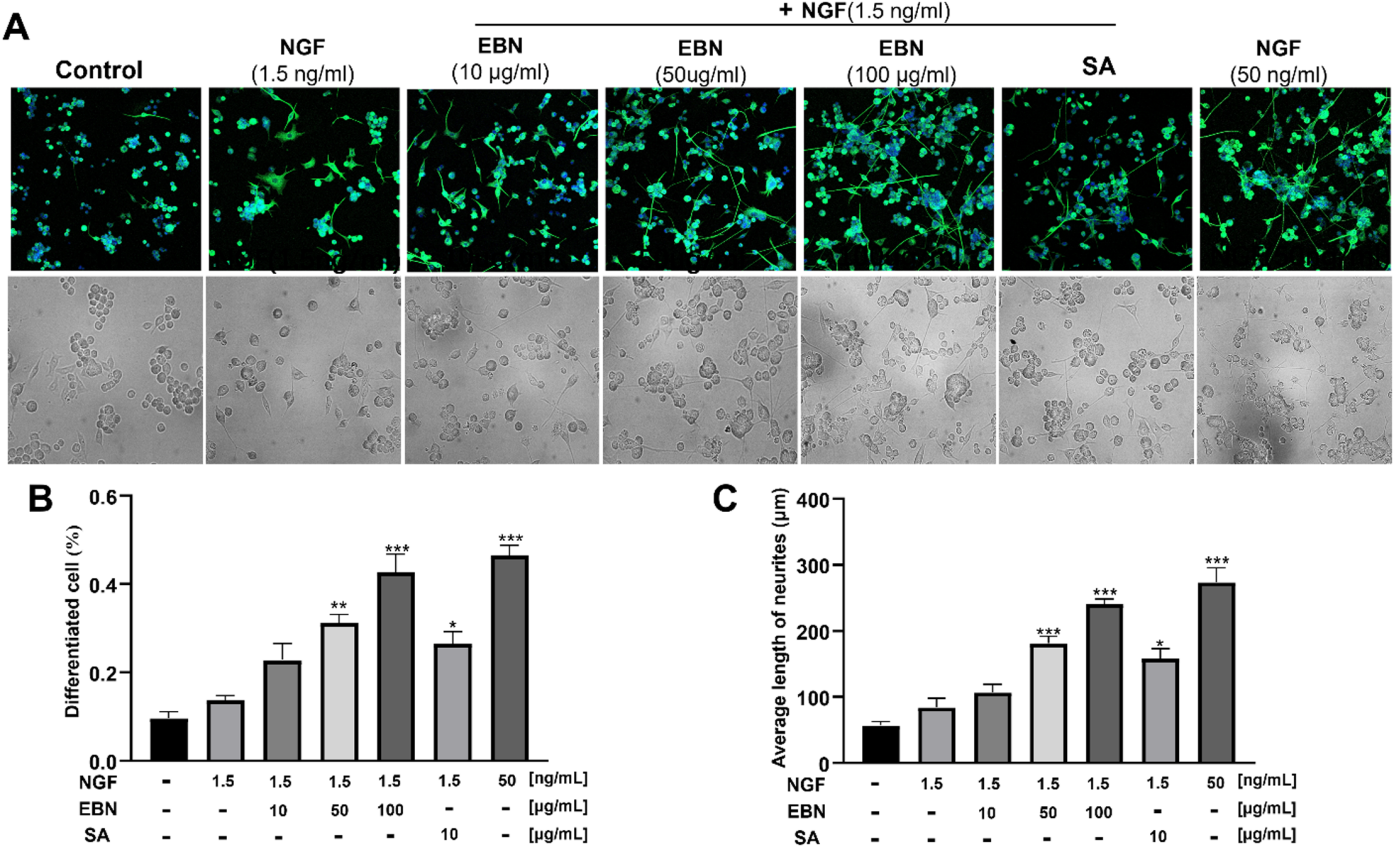
EBND enhances NGF‐induced neural synapse growth in PC12 cells by co‐treatment. (A) Representative immunofluorescence images of PC‐12 cells treated with EBND (μg/mL) in the presence of trace amounts of NGF for 7 days. Cytoskeletal β‐III‐tubulin proteins (green) were used to depict differences in neurite length between treatment groups. (B, C) Quantitative results of number of differentiated cells and average neurite lengths. Data are presented as mean ± SEM, *n* ≥ 3. (*p < 0.05, **p < 0.01, ***p < 0.001).

**FIGURE 4:**
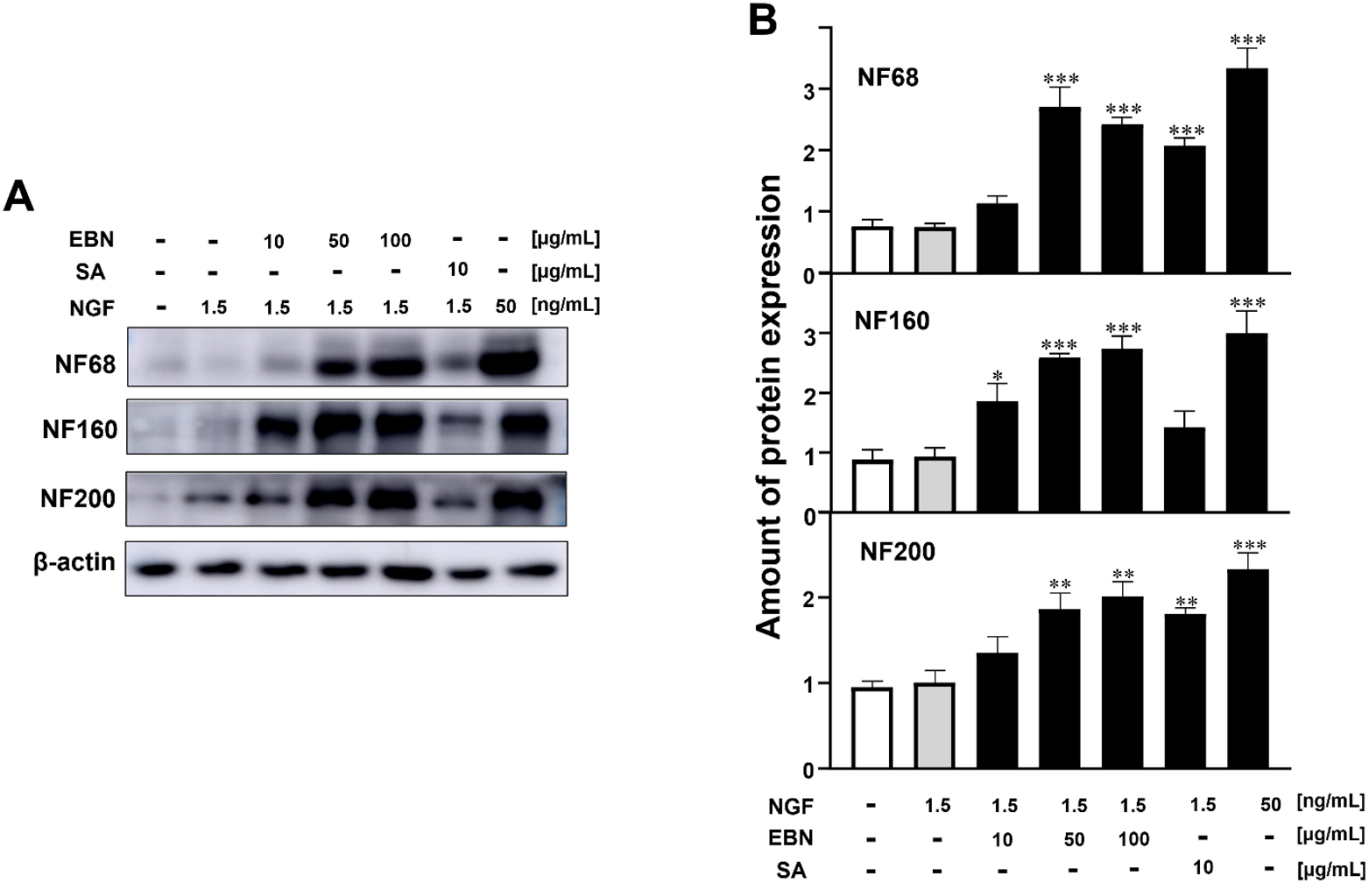
EBND potentiate the NGF‐induced expression of neurofilaments. (A, B) PC‐12 cells were treated with different concentrations of EBND (μg/mL) for 7 days in the presence of trace amounts of NGF, and protein expression of neurofilament NF68, NF160, and NF200 was detected by western blot. 50 ng/mL NGF was used as a positive control. Data are presented as mean ± SEM, *n* ≥ 3. (*p < 0.05, **p < 0.01, ***p < 0.001).

### 3.3 EBND Activates the TrkA‐ERK1/2‐CREB‐BDNF Signaling Pathway

To elucidate the processes underlying the EBND‐induced differentiation of PC12 cells, we examined changes in the TrkA signaling pathway. NGF triggers neuronal differentiation and enhances cell viability by activating TrkA receptors in PC12 cells (Xiong Gao et al. 2021). The effect of EBND on TrkA phosphorylation was also examined. TrkA phosphorylation in PC12 cells treated with 1.5 ng/mL NGF alone was minimal. Notably, TrkA phosphorylation was significantly increased, by more than 2‐fold, after co‐treatment with EBND (*p* = 0.003) (Figure 5A). Moreover, TrkA phosphorylation was increased 1.5‐fold in the SA group compared with the 1.5 ng/mL NGF‐treated control group (*p* = 0.02) (Figure 5A). Notably, BDNF was upregulated by 44.8% in the EBND compared with the 1.5 ng/mL NGF group (*p* = 0.2309) (Figure 5A). Next, we examined the expression of TrkA's downstream effectors, extracellular signal‐regulated kinases (Erk1/2) and the cAMP response element‐binding protein (CREB). The group given NGF and EBND co‐treatment showed a 3‐fold increase in ERK1/2 phosphorylation (*p* < 0.001) and a 2‐fold increase in CREB phosphorylation (*p* = 0.02), compared with the control group treated with 1.5 ng/mL NGF (Figure 5B,C). Furthermore, the SA group showed significantly increased phosphorylation levels of ERK1/2 (*p* = 0.03) and CREB (*p* = 0.01) (Figure 5B,C). These findings suggest that signaling pathways transduce and relay the NGF signal at distal axons to promote the activation of ERK1/2 and the phosphorylation of CREB in the nucleus. Phosphorylated CREB (p‐CREB) in turn induces expression of BDNF, which is critical for neurogenesis and neuronal differentiation (Réus et al. 2011). Based on these findings, we posit that EBND potentiates NGF‐induced TrkA phosphorylation, which in turn activates CREB through the ERK1/2 signaling cascade, culminating in a significant increase in BDNF expression, which is pivotal for neuronal differentiation.

**FIGURE 5:**
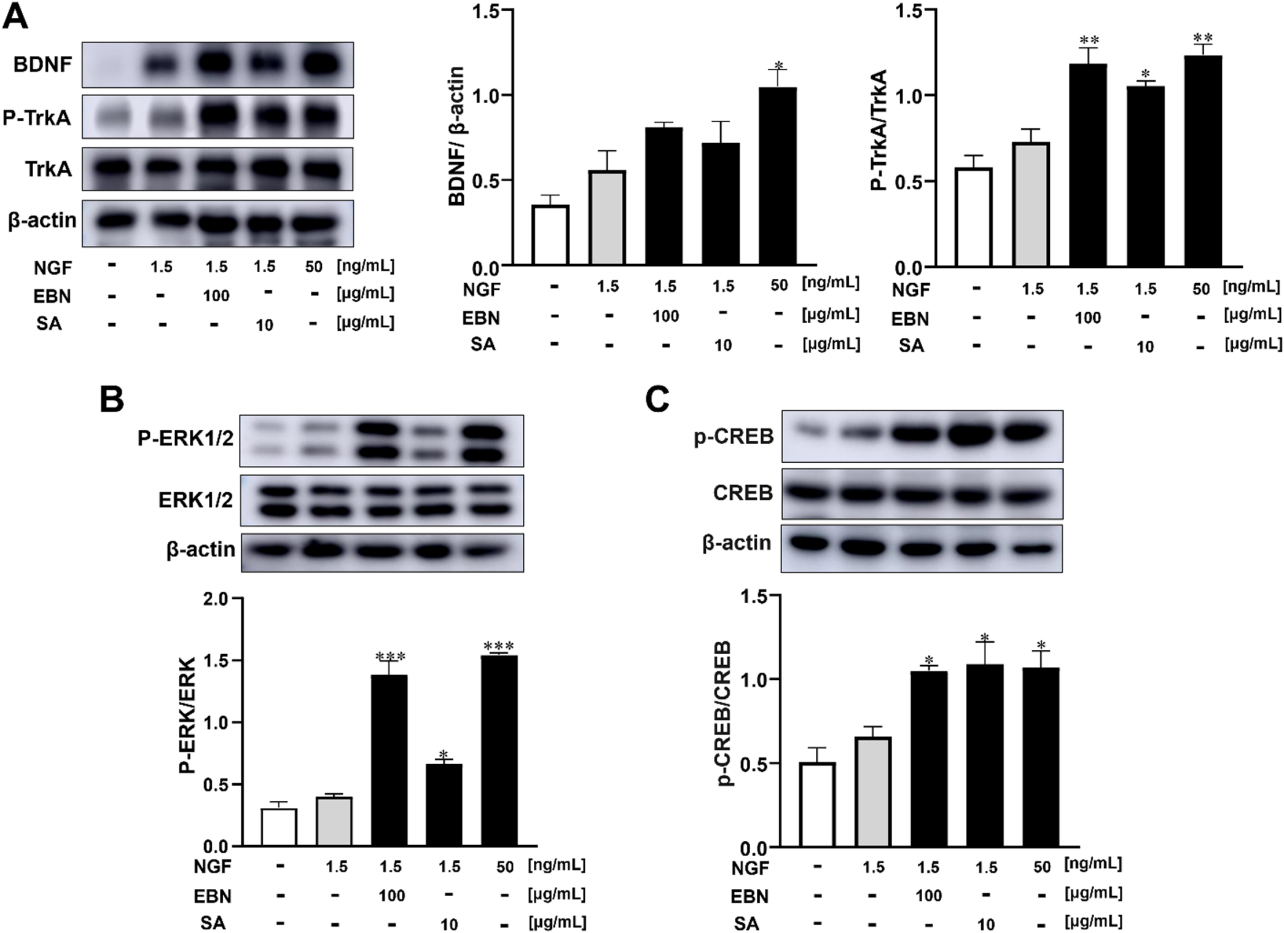
EBND co‐treatment with NGF promotes phosphorylation of TrkA, ERK1/2, CREB and protein expression of BDNF in PC12 cells. Protein expression levels of BDNF and p‐TrkA (A), p‐CREB (B) and p‐ERK1/2 (C) in the cells from each experimental group were assessed using western blot analysis after 48 h of treatment, followed by quantitative analysis. Data are presented as mea*n* ± SEM, *n* ≥ 3. (*p < 0.05, **p < 0.01, ***p < 0.001).

### 3.4 Analysis of the TrkA‐ERK1/2‐CREB‐BDNF Signaling Pathway

To examine whether EBND exerts its effects by activating TrkA receptors and triggering the ERK1/2 signaling pathway, we treated PC12 cells with 1.5 ng/mL NGF along with EBND, either with or without the concurrent administration of the inhibitor GW441756 at 100 nM. Following a 48‐h treatment period, we extracted cellular proteins to assess neurofilament levels. As shown in Figure 6, there was a marked reduction in the levels of NF68 (*p* = 0.03), NF160 (*p* = 0.01), and NF200 (*p* = 0.01) following the application of GW441756 (Figure 6A,B). These results suggest that EBND promotes neuronal differentiation by upregulating TrkA, thereby enhancing the sensitivity of cells to low concentrations of NGF.

**FIGURE 6:**
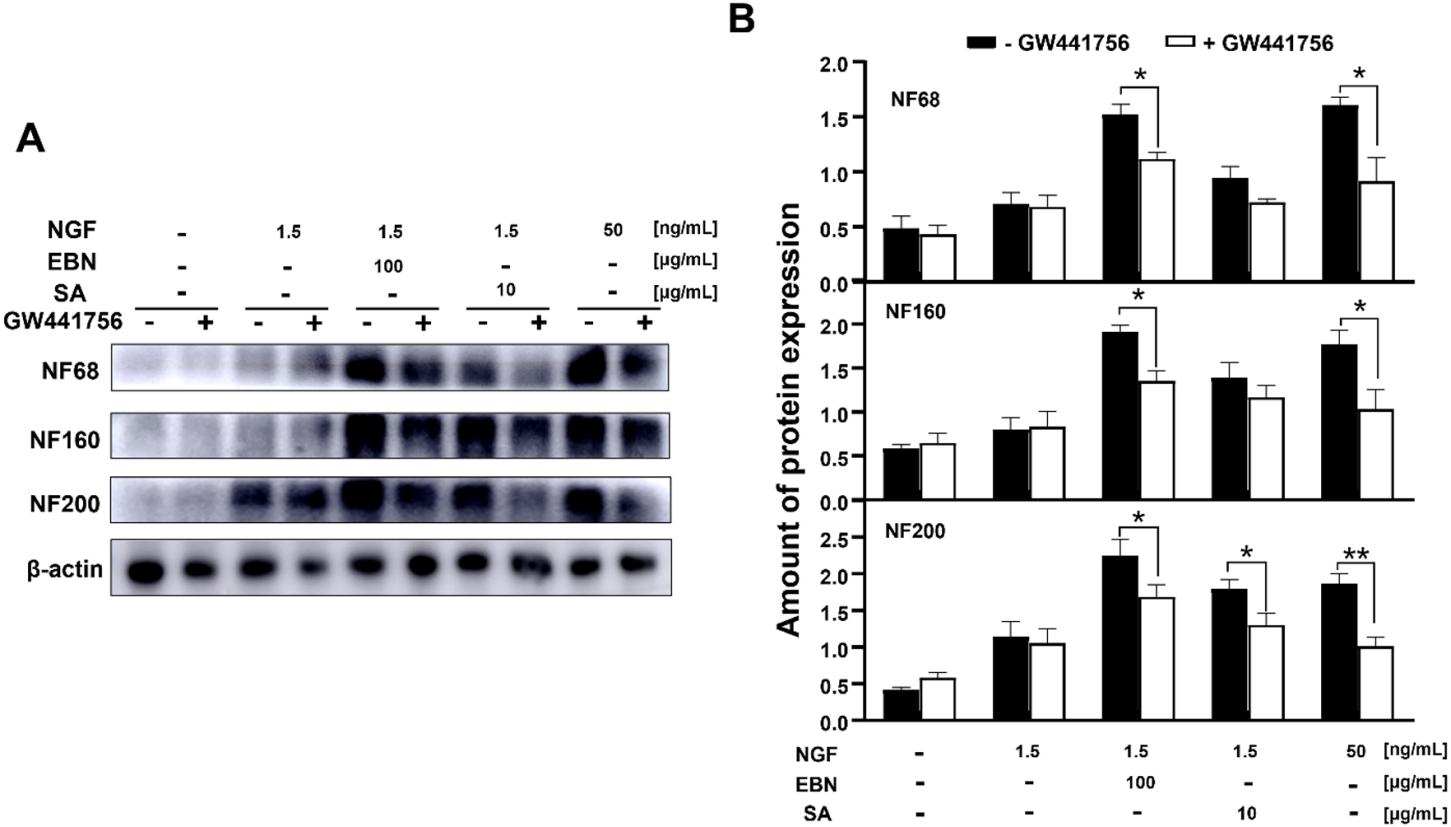
Validation of the EBND neurogenic action signaling pathway. PC12 cells were pretreated with 100 nM GW441765 for 1 h before treating the cells with the drug. (A, B) The expression levels of neurofilament proteins were determined by Western blot (*p < 0.05, **p < 0.01). Data are presented as mea*n* ± SEM (*n* ≥ 3).

## 4 Discussion

Neurogenesis, neuronal differentiation and growth are crucial for brain development. The growth of neurites is a pivotal step in neuronal development, and the loss of neurites is a key feature of neuronal damage (More et al. 2012). Neuronal loss and damage not only critically shape cognitive development in childhood, but also have profound implications for aging‐related neurodegenerative disorders, such as Alzheimer, Parkinson and Huntington diseases (Schengrund 2015). Promoting neurogenesis may therefore enhance recovery from neurodegenerative diseases (Costa et al. 2015). An increasing number of researchers are investigating approaches to improve memory through the enhancement of neuronal differentiation and the mitigation of neuronal damage. EBN supplementation in pregnant and lactating female mice has been shown to enhance neonatal learning and memory performance, likely by upregulating BDNF, and thereby promoting neurogenesis in the hippocampus (Mahaq et al. 2020; Xie et al. 2018). Therefore, EBN is considered to have beneficial effects on cognitive function. Our previous animal studies demonstrated that EBN exerts a cognitive‐enhancing effect, which is potentially linked to the promotion of neurogenesis and synaptic plasticity (Zhu et al. 2023). However, the molecular mechanisms that promote neurogenesis remain to be explored. Building on these previous studies, we employed the PC12 cell line in the present study to investigate the mechanisms of neuronal differentiation.

Neuronal differentiation relies on the individual or synergistic action of extracellular neurotrophic factors that regulate the development, regeneration and function of the nervous system (Sultan et al. 2021). PC12 cells are extensively used as a versatile model system for neurobiological and neurotoxicological studies because of their similarity to sympathetic neurons. PC12 cells can divide rapidly, grow in suspended aggregates, and exhibit pronounced sensitivity to nerve growth factor (NGF). Exposure to this neurotrophic factor induces a cessation of cell proliferation, which is followed by significant phenotypic changes as the cells acquire typical neurite extensions and characteristics of sympathetic neurons. Here, we used PC12 cells to investigate the effects of EBN on neuronal differentiation by measuring neurite length and counting the number of differentiated cells (Oda et al. 2007; Chen et al. 2009). Additionally, neuronal differentiation can be assessed using biomarkers such as neurofilament expression. Neurofilaments, major components of the neuronal cytoskeleton, have molecular weights of approximately 68 kDa, 160 kDa and 200 kDa. NF68 and NF160 are typically expressed during the early stages of neuronal differentiation, whereas NF200 is expressed in neurons that have reached a mature stage of differentiation (Carden et al. 1987). Our results demonstrate that morphologically, treatment of PC12 cells with EBND did not result in directly observable neurite outgrowth, but it did elevate the expression levels of the three neurofilament proteins. However, both neurite growth and neurofilament protein expression were significantly enhanced in the group given combined EBND and low‐dose NGF treatment. Therefore, EBND has the ability to enhance NGF‐induced neurite growth.

In the brain, neurotrophic factors are endogenous molecules that play critical roles in neuronal development (Urbán and Guillemot 2014). NGF is a key regulator of neurogenesis, synaptic growth, neuroplasticity, and axonal growth during neuronal development. Furthermore, it plays an important role in regulating normal cognitive functions such as learning, memory, and sensory perception. Notably, many neurodegenerative disorders, including depression and Alzheimer disease, have been linked closely to a deficiency of NGF in the brain (Xu et al. 2016, 2013). Trk receptors influence the survival and differentiation of neurons via many signaling cascades. Neurotrophin interaction with Trk receptors triggers a signaling cascade involving PLC, PI3K/Akt, and Ras/MAPK signaling pathways (Dahiya et al. 2025). It has been reported that binding of natural products, such as lignans and pterostilbene, to NGF may enhance the interaction between NGF and its high‐affinity receptor, TrkA. This could potentially promote neuronal differentiation and neurite outgrowth through (Xiong Gao et al. 2021; Ospondpant et al. 2025). The activation of the ERK/CREB signaling pathway. In this study, in the presence of low concentrations of NGF, treatment with EBND markedly increased the phosphorylation of TrkA and ERK. Notably, levels of p‐ERK1/2 were significantly increased, in turn activating CREB. The ensuing high levels of p‐CREB induced expression of BDNF (Motaghinejad et al. 2017; Esvald et al. 2020). This enhancement was blocked by the TrkA inhibitor GW441756, confirming that EBND mediates its effects via TrkA. Together, these findings suggest that EBND functions as a neurotrophic factor that augments NGF signaling and stimulates neurite growth via the TrkA‐ERK1/2‐CREB‐BDNF pathway in PC12 cells. The mechanism of action is shown in Figure 7.

**FIGURE 7:**
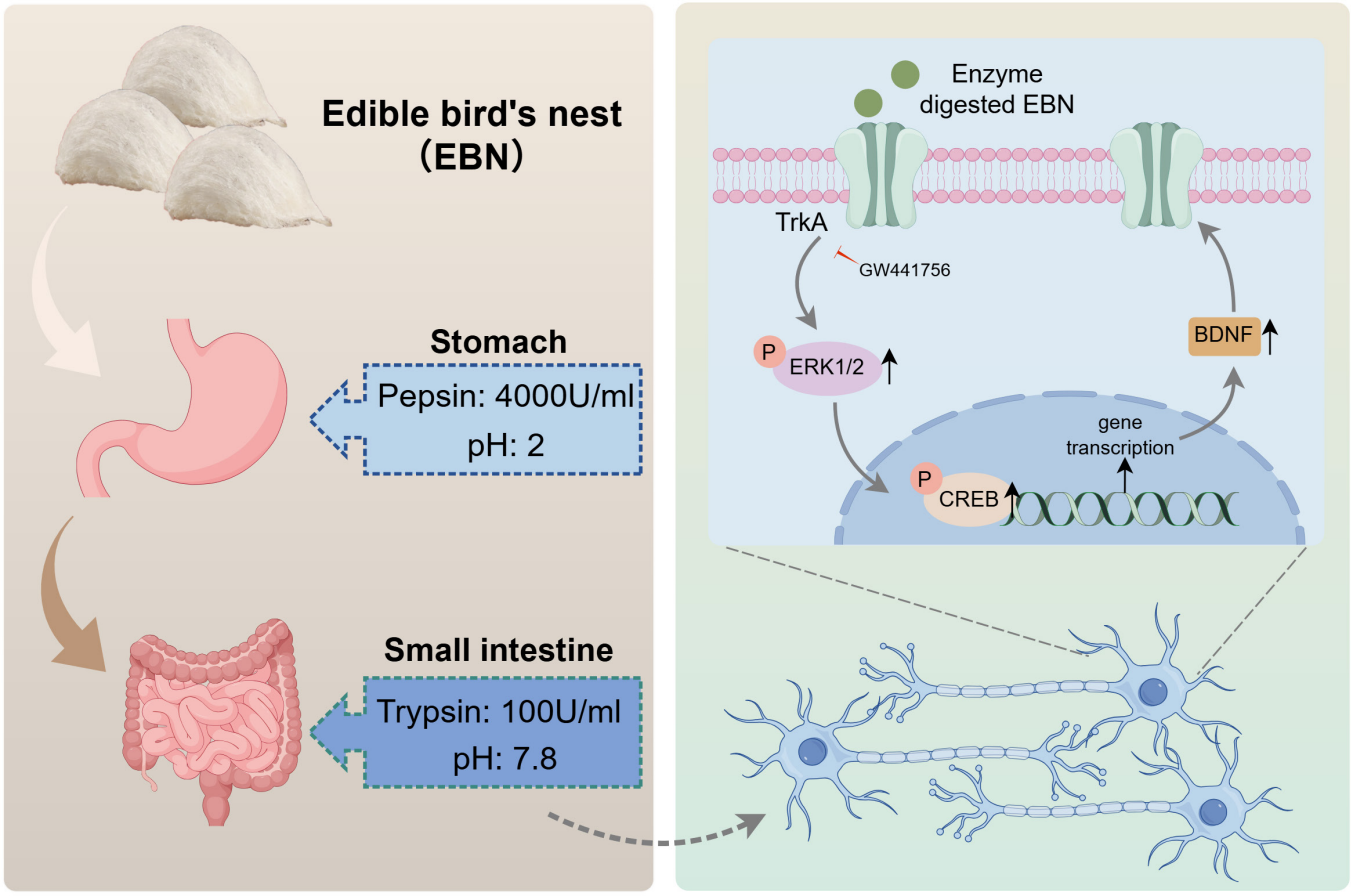
Potential mechanisms by which EBND promotes NGF‐induced differentiation of PC12 cells. The black arrows indicate the upregulated function by EBND. This figure was drawn using Figdraw software.

An interesting observation in our study was that EBND treatment alone significantly increased the expression of neurofilament proteins (NF68, NF160, NF200) without concurrently inducing robust neurite outgrowth (Figure 2). Neurofilaments are key structural components of the neuronal cytoskeleton and their upregulated expression is a well‐established early biochemical marker of neuronal differentiation commitment, often preceding or occurring independently of extensive morphological changes (Carden et al. 1987; Kotaich et al. 2023). The increase in NF68 and NF160, typically associated with early differentiation stages, suggests that EBND initiates the molecular program of neuronal differentiation. However, the absence of strong neurite growth under EBND‐alone treatment implies that the complete morphological manifestation of differentiation—characterized by neurite elongation—may require additional signaling cues or a permissive cellular context. This is supported by our key finding that in the presence of a suboptimal dose of NGF (which alone is insufficient to trigger differentiation), EBND potently synergizes to elicit both significant neurofilament expression and robust neurite extension (Figures 3 and 4). This indicates that EBND likely primes the cells by upregulating differentiation‐related proteins like neurofilaments, while NGF provides the essential trophic support to translate this molecular priming into structural growth via pathways like TrkA‐ERK1/2‐CREB‐BDNF. Therefore, the neurofilament upregulation by EBND alone represents a state of “differentiation priming,” setting the stage for enhanced responsiveness to co‐existing neurotrophic signals like NGF.

However, this study has several limitations that should be considered. Firstly, our conclusions are based on findings from the PC12 cell line model. Future validation in other neuronal cell models is warranted to assess the broader applicability of these results. Secondly, EBND is a complex enzymatic hydrolysate. Our findings support that sialic acid (SA) contributes to its neurogenic properties by enhancing TrkA‐ERK1/2‐CREB‐BDNF signaling, which aligns with previous reports on SA's role in brain development and cognition (Wang 2009). However, EBND elicited markedly stronger effects than an equivalent amount of purified SA, indicating that its neurogenic activity cannot be attributed to SA alone. This underscores the complexity of EBND, where other bioactive constituents (e.g., EGF‐like molecules, or specific peptides from enzymatic digestion) may exert independent or synergistic effects. Further in‐depth exploration of the functional components of EBN is needed in the future. Thirdly, certain limitations also exist regarding the digestion method used for the bird's nest. Boiling (95°C–100°C) was employed to terminate enzymatic hydrolysis by inactivating pepsin and trypsin. While common in the literature, this heat treatment may degrade some heat‐sensitive components. Future studies could explore alternative termination methods (e.g., protease inhibitors plus rapid freezing) to better preserve the product's compositional and bioactive profile. Lastly, the observation that EBND alone upregulated early differentiation markers (neurofilament proteins) but did not robustly promote morphological neurite outgrowth suggests its role may be as a “differentiation primer”, with complete morphological maturation potentially requiring synergistic signals. Our study did not fully elucidate the underlying reasons for this dissociation between molecular and morphological changes. Future research should focus on identifying the key active components within EBND and further investigating the temporal dynamics of its priming effects on neuronal differentiation.

## 5 Conclusion

In conclusion, memory impairment may result from a deficiency of neurotrophic factors. Our findings suggest that EBND may potentiate NGF signaling to promote the differentiation and growth of neurons. This effect appears to be mediated by the TrkA‐ERK1/2‐CREB‐BDNF signaling cascade. Our findings provide insight into the neuroprotective mechanisms of action of EBND, and they suggest that EBN, a safe and functional natural food, may have application in the development of dietary supplements for neurodegenerative diseases related to NGF deficiency. However, additional studies are needed to clarify the underlying mechanisms, including how EBND enhances NGF signaling. In addition, the bioactive components in EBND that mediate these actions remain unknown. We found that SA is far less potent than EBND, suggesting that other components in EBND underlie the neuroprotective effects. Further studies are needed to identify the key bioactive factors in EBND.

## Author Contributions

**Wen Zhang:** writing – original draft, data curation, writing – review and editing. **Xintong Wang:** writing – original draft, writing – review and editing, data curation. **Huiqing Zhu:** resources. **Xiaoxian Lin:** funding acquisition. **Rongzi Li:** visualization. **Man Yuan:** funding acquisition. **Baocai Xu:** supervision. **Dongliang Wang:** resources, visualization. **Wei Xiong:** conceptualization, methodology. **Yixuan Li:** validation, project administration.

## Funding

This work was supported by 111 project from the Education Ministry of China, No. B18053. Major cooperation projects between Chinese Academy of Engineering and local authorities, 2023HENZDA02.

## Ethics Statement

This study does not involve any human or animal testing.

## Conflicts of Interest

The authors declare no conflicts of interest.

## Supporting information


**Figure S1:** Effect of EBND on PC12 cell viability. PC12 cells were treated with various concentrations of EBND (1, 10, 100, 500, 1000 μg/mL) for indicated time, and cell viability was measured by CCK‐8 assay. Data are presented as mean ± SEM, *n* = 4. (*p < 0.05, **p < 0.01 compared with control group).
**Table S1:** Original data.
**Table S2:** Summary of one‐way ANOVA statistics for all experiments.

## Data Availability

The data that support the findings of this study are available from the corresponding author upon reasonable request.
