# Supplementary material for: Enzyme‐Digested Edible Bird's Nest Promotes NGF‐Induced Differentiation of PC12 Cells by Activating the TrkA‐ERK1/2‐CREB‐BDNF Signaling Pathway
Source: Food Sci Nutr. 2026 Oct 6;14(10):e72419. doi: 10.1002/fsn3.72419 (PMC13643363; doi:10.1002/fsn3.72419)
Supplement: Supplementary file 1 [file FSN3-14-e72419-s001.docx]

**Supplementary Figures**

**Figure S1**

**Figure S1**. Effect of EBND on PC12 cell viability. PC12 cells were treated with various concentrations of EBND (1, 10, 100, 500, 1000 μg/mL) for indicated time, and cell viability was measured by CCK-8 assay. Data are presented as mean ± SEM, n =4. (**P* < 0.05, ***P* < 0.01 compared with control group).

**Table S1 original data**

| **Concentration (μg/mL)** | **Replicate 1** | **Replicate 2** | **Replicate 3** | **Replicate 4** | **Mean ± SEM** |
| --- | --- | --- | --- | --- | --- |
| **Control** | 96.13 | 100.08 | 99.09 | 104.69 | 100.00 ± 1.78 |
| **1** | 97.45 | 111.28 | 108.31 | 113.25 | 107.57 ± 3.52 |
| **10** | 111.93 | 106.34 | 103.7 | 118.52 | 110.12 ± 3.28 |
| **100** | 118.85 | 112.26 | 112.26 | 117.20 | 115.14 ± 1.70 |
| **500** | 112.59 | 114.57 | 116.21 | / | 114.46 ± 1.05 |
| **1000** | 111.6 | 109.30 | 102.39 | / | 107.76 ± 2.77 |

**Supplementary Table S2. Summary of one-way ANOVA statistics for all experiments.**

| **Figure** | **Dependent variable** | **F (DFn, Dfd)** | **Significant post hoc comparisons** |
| --- | --- | --- | --- |
| Fig. S1 | Cell Viability (100%) | F (5, 16) = 4.62 | Control vs 1 ug/mL EBND: *P* = 0.17, ns  Control vs 10 ug/mL EBND: *P* = 0.05, *  Control vs 100 ug/mL EBND: *P* = 0.003, **  Control vs 500 ug/mL EBND: *P* = 0.007, **  Control vs 1000 ug/mL EBND: *P* = 0.21, ns |
| Fig. 1B | Differentiated cell (%) | F (5, 18) = 80.01 | 0 vs 10 ug/mL EBND: *P* = 0.80, ns; 0 vs 50 ug/Ml EBND: *P =* 0.72, ns; 0 vs 100 ug/mL EBND: *P* = 0.11, ns; 0 vs 10 ug/ mL SA: *P* = 0.61, ns; 0 vs 50 ug/ mL NGF: *P* < 0.001, *** |
| Fig. 1C | Average length of neurites (um) | F (5, 53) = 97.90 | 0 vs 10 ug/mL EBND: *P* > 0.99, ns; 0 vs 50 ug/Ml EBND: *P =* 0.36, ns; 0 vs 100 ug/mL EBND: *P* = 0.27, ns; 0 vs 10 ug/ mL SA: *P* = 0.92, ns; 0 vs 50 ug/ mL NGF: *P* < 0.001, *** |
| Fig. 2B | NF68/β-actin | F (5, 11) = 139.6 | 0 vs 10 ug/mL EBND: *P* > 0.99, ns; 0 vs 50 ug/Ml EBND: *P <* 0.001, ***; 0 vs 100 ug/mL EBND: *P* < 0.001, ***; 0 vs 10 ug/ mL SA: *P* < 0.001, ***; 0 vs 50 ug/ mL NGF: *P* < 0.001, *** |
| Fig. 2B | NF160/β-actin | F (5, 12) = 17.88 | 0 vs 10 ug/mL EBND: *P* > 0.99, ns; 0 vs 50 ug/mL EBND: *P =* 0.65, ns; 0 vs 100 ug/mL EBND: *P* = 0.003, **; 0 vs 10 ug/mL SA: *P* = 0.96, ns; 0 vs 50 ug/mL NGF: *P* < 0.001, *** |
| Fig. 2B | NF200/β-actin | F (5,12) = 32.13 | 0 vs 10 ug/mL EBND: *P* > 0.99, ns; 0 vs 50 ug/mL EBND: *P =* 0.83, ns; 0 vs 100 ug/mL EBND: *P* = 0.01, *; 0 vs 10 ug/mL SA: *P* = 0.34, ns; 0 vs 50 ug/mL NGF: *P* < 0.001, *** |
| Fig. 3B | Differentiated cells (%) | F (6, 19) = 23.87 | 1.5 ng/mL NGF vs 10 ug/mL: *P* = 0.12, ns  1.5 ng/mL NGF vs 50 ug/mL: *P* = 0.002, **  1.5 ng/mL NGF vs 100 ug/mL: *P* < 0.001, ***  1.5 ng/mL NGF vs SA: *P* = 0.02, *  1.5 ng/mL NGF vs 50 ng/mL EBND: *P* < 0.001, *** |
| Fig. 3C | Average neurite length (μm) | F (6, 41) = 28.19 | 1.5 ng/mL NGF vs 10 ug/mL EBND: *P* = 0.797, ns  1.5 ng/mL NGF vs 50 ug/mL EBND: *P* < 0.001, ***  1.5 ng/mL NGF vs 100 ug/mL EBND: *P* < 0.001, ***  1.5 ng/mL NGF vs SA: *P* = 0.016, *  1.5 ng/Ml NGF vs 50 ng/mL NGF: *P* < 0.001, *** |
| Fig. 4B | NF68/β-actin | F (6, 14) = 31.16 | 1.5 ng/mL NGF vs 10 ug/mL EBND: *P* = 0.51, ns  1.5 ng/mL NGF vs 50 ug/mL EBND: *P* < 0.001, ***  1.5 ng/mL NGF vs 100 ug/mL EBND: *P* < 0.001, ***  1.5 ng/mL NGF vs SA: *P* = 0.001, ***  1.5 ng/mL NGF vs 50 ng/mL NGF: *P* < 0.001, *** |
| Fig. 4B | NF160/β-actin | F (6, 14) = 17.54 | 1.5 ng/mL NGF vs 10ug/mL EBND: *P* = 0.03, *  1.5 ng/mL NGF vs 50 ug/mL EBND: *P* < 0.001, ***  1.5 ng/mL NGF vs 100 ug/mL EBND: *P* < 0.001, ***  1.5 ng/mL NGF vs SA: *P* = 0.40, ns  1.5 ng/mL NGF vs 50 ng/mL NGF: *P* < 0.001, *** |
| Fig. 4B | NF200/β-actin | F (6, 14) = 12.82 | 1.5 ng/mL NGF vs 10 ug/mL EBND: *P* = 0.40, ns  1.5 ng/mL NGF vs 50 ug/mL EBND: *P* = 0.005, **  1.5 ng/mL NGF vs 100 ug/mL EBND: *P* = 0.001, **  1.5 ng/ml NGF vs SA: *P* = 0.008, **  1.5 ng/mL NGF vs 50 ng/mL EBND: *P* < 0.001, *** |
| Fig. 5A | p-TrkA/TrkA | F (4, 10) = 17.79 | 1.5 ng/mL NGF vs 100 ug/mL EBND: *P* = 0.003, **  1.5 ng/mL NGF vs SA: *P* =0.02, *  1.5 ng/mL NGF vs 50 ng/mL NGF: *P* = 0.001, ** |
| Fig. 5A | BDNF/β-actin | F (4, 10) = 7.973 | 1.5 ng/mL NGF vs 100 ug/ mL EBND: *P* = 0.2309, ns  1.5 ng/mL NGF vs SA: *P* =0.5839, ns  1.5 ng/mL NGF vs 50 ng/mL NGF: *P* = 0.0125, * |
| Fig. 5B | p-ERK1/2/ERK1/2 | F (4, 10) = 98.94 | 1.5 ng/mL NGF vs 100 ug/ mL EBND: *P* < 0.001, ***  1.5 ng/mL NGF vs SA: *P* =0.03, *  1.5 ng/mL NGF vs 50 ng/mL NGF: *P* < 0.001, *** |
| Fig. 5C | p-CREB/CREB | F (4, 15) = 9.467 | 1.5 ng/mL NGF vs 100 ug/ mL: *P* = 0.02, *  1.5 ng/mL NGF vs SA: *P* =0.01, *  1.5 ng/mL NGF vs 50 ng/mL NGF: *P* < 0.02, * |
| Fig. 6B | NF68/β-actin | F (4, 20) = 3.185 | 1.5 ng/mL NGF+100 ug/ mL EBND vs 1.5 ng/mL NGF+100 ug/ mL EBND+GW441756: *P* = 0.03, *;  50 ng/mL NGF vs 50 ng/mL NGF +GW441756: *P* = 0.04, *; |
| Fig. 6B | NF160/β-actin | F (4, 20) = 2.968 | 1.5 ng/mL NGF+100 ug/ mL EBND vs 1.5 ng/mL NGF+100 ug/ mL EBND+GW441756: *P* = 0.01, *;  50 ng/mL NGF vs 50 ng/mL NGF +GW441756: *P* = 0.03, * |
| Fig. 6B | NF200/β-actin | F (4, 20) = 3.200 | 1.5 ng/mL NGF+100 ug/ mL EBND vs 1.5 ng/mL NGF+100 EBND+GW441756: *P* = 0.01, *;  1.5 ng/mL NGF + SA vs 1.5 ng/mL NGF + SA+ GW441756: *P* = 0.04, *  50 ng/mL NGF vs 50 ng/mL NGF +GW441756: *P* = 0.004, ** |
